# Association of genetic variations in *FoxP3* gene with Graves' disease in a Southwest Chinese Han population

**DOI:** 10.1002/iid3.1046

**Published:** 2023-10-13

**Authors:** Guiqin Tan, Guangbing Zheng, Jiang Li, Yingping Zhu, Zhongzhi Liang, Hua Li, Hongsong Yu, Xin Wang

**Affiliations:** ^1^ School of Basic Medical Sciences, Special Key Laboratory of Ocular Diseases of Guizhou Province Zunyi Medical University Zunyi China; ^2^ The Second Affiliated Hospital of Zunyi Medical University Zunyi China; ^3^ Yongchuan Hospital Chongqing Medical University Chongqing China

**Keywords:** association, Forkhead box P3, Graves' disease, single‐nucleotide polymorphism

## Abstract

**Background:**

Graves' disease (GD) is a T cell‐mediated organ‐specific autoimmune disease. Forkhead box P3 (FoxP3) is an excellent marker for the induction and development of regulatory T cells (Tregs). Recent studies showed that single‐nucleotide polymorphisms (SNPs) in the *FoxP3* gene were associated with the increased susceptibility to several autoimmune diseases. In the present study, we investigated the association of *FoxP3* gene polymorphisms with GD in a Southwest Chinese Han population.

**Methods:**

A two‐stage case‐control study was performed in 890 healthy controls (male, 282; female, 608) and 503 patients with GD (male, 138; female, 365). Four SNPs (rs3761548, rs3761549, rs3761547, and rs2280883) were genotyped by the polymerase chain reaction‐restriction fragment length polymorphism assay. The *χ* ^2^ test was used to compare the genotype distributions and allele frequencies between GD patients and healthy controls.

**Results:**

In the first stage, the significantly increased frequencies of the A allele (*p* = .031, odds ratio [OR] = 1.635) and AA genotype (*p* = .023, OR = 3.257), together with a significantly decreased frequency of the C allele (*p* = .031, OR = 0.611) of *F*ox*P3*/rs3761548 were found in female patients with GD. None of the other *FoxP3* SNPs was associated with GD susceptibility. Subsequent validation and combination of data confirmed the association between *FoxP*3/rs3761548 and the female patients with GD (A allele: *p* < .001, OR = 1.672; AA genotype: *p* = .005, OR = 2.488; CC genotype: *p* = .001, OR = 0.622; C allele: *p* < .001, OR = 0.615, respectively).

**Conclusion:**

Our findings suggest that *F*ox*P*3/rs3761548 is significantly associated with female GD patients in a Southwest Chinese Han population.

## 1 INTRODUCTION

Graves' disease (GD), also known as toxic hyperthyroidism, is the most common cause of hyperthyroidism. It is an autoimmune disease caused by thyrotropin receptor antibody produced by the thyroid‐stimulating hormone receptor. GD is also a multisystem syndrome charactered by hypermetabolic syndrome, diffuse goiter, eye sign, skin lesions, and thyroid acromegaly. GD is the most common in patients between the ages of 20 and 40 year, with a male: female prevalence of 1:4−1:6.^1^ The annual incidence rate is approximately 20−30/1,000,000.^2^ Although the pathogenesis of GD has not been fully elucidated, it is believed to be the result of the interaction between genetic susceptibility and environmental factors.^3^, ^4^ Therefore, the identification of genes and sites contributing to GD susceptibility is an important issue in the study of the pathogenesis of this disease.

Regulatory T cells (Tregs) play a pivotal role not only in the suppression of immune responses and the development of immune tolerance but also in balancing immune responses by suppressing immune hyper‐reactivity through the secretion of inhibitory cytokines or direct cell contact‐dependent mechanisms.^5^ Th17/Treg homeostasis is disrupted in many autoimmune diseases.^6^ Previous studies showed functional defects in Tregs and increased numbers of Th17 cells in GD patients,^7^, ^8^ while another study showed that Treg levels were reduced in GD patients compared with healthy controls, suggesting that Tregs play an important role in the pathogenesis of GD.^9^

Forkhead box P3 (FoxP3) is a transcription factor specifically and stably expressed in Tregs. The *FoxP3* gene is located on human chromosome Xp11.23. Polymorphisms in *FoxP3* may change its expression level and weaken the inhibitory function of Tregs, thus affecting the role of Tregs in the immune response and potential autoimmune diseases.^10^ The rs3761547, rs3761548, rs3761549, and rs2280883 single‐nucleotide polymorphisms (SNPs) are related to *FoxP3* expression.^11^ The role of *FoxP3* polymorphisms in the development of GD is not well understood. Independent studies in China, Poland, India, and Japan have shown that certain *FoxP3* mutations are associated with GD susceptibility.^12^, ^13^, ^14^, ^15^, ^16^ However, another study found there was no association between *FoxP3* polymorphism and GD susceptibility in United Kingdom population.^17^

Although previous studies showed that *FoxP3* polymorphism plays a critical role in the pathogenesis of GD, the results are inconsistent due to differences in environmental conditions or genetic backgrounds. Study by Zheng et al.^13^ on *FoxP3* gene polymorphisms were associated with GD susceptibility among Han population in central plains of China. However, to date, there has been no comprehensive investigation into the association between *FoxP3* polymorphisms and GD susceptibility in the Southwest Chinese Han population. Therefore, we explored whether the *FoxP3* SNPs (rs3761548, rs3761549, rs3761547, and rs2280883) were associated with GD susceptibility in a Southwest Chinese Han population.

## 2 METHODS

### 2.1 Study subjects

A total of 503 GD patients and 890 healthy controls were recruited from the Affiliated Hospital of Zunyi Medical University and the Yongchuan Hospital of Chongqing Medical University. The cases and controls were recruited from January 2016 to December 2020. All patients were diagnosed according to the 2016 Guidelines of the American Thyroid Association and the Society of Clinical Endocrinologists for the Diagnosis and Treatment of Hyperthyroidism.^18^ The controls were selected from the health examination centers of these two hospitals, and they did not have any other autoimmune disease and were matched for sex and age with the patients with GD. This study was also registered in the Chinese Clinical Trial Registry (registration number: ChiCTR1900022398). The written informed consent was obtained from all participants in the study before blood collection.

### 2.2 DNA extraction

Venous blood samples were collected from patients with GD and healthy controls into EDTA anticoagulant tubes. Genomic DNA was extracted from peripheral blood using the QIAamp DNA Blood Mini Kit (Qiagen). The purity of DNA was determined according to the optical density ratio of 260/280 nm.

### 2.3 Genotyping

The polymerase chain reaction‐restriction fragment length polymorphism assay was conducted to genotype the four SNPs (rs3761547, rs3761548, rs3761549, and rs2280883) of *FoxP3* gene, which were established to be associated with autoimmune diseases in previous studies. The target sequence was amplified by PCR with suitable primers.^13^ The target sequence of each gene was amplified by PCR and the product was digested with specific restriction enzymes. Table 1 summarizes the sequences of the forward and reverse primers, PCR conditions and restriction enzymes. Five samples were randomly repeated for genotyping, and the results were the same as before.

**Table 1:** Primers, reaction conditions and restriction enzymes used for PCR‐RFLP analysis.

SNP	Primer	Reaction conditions of PCR	Restriction enzyme	Product size
rs3761549	5′‐GCCTGGCACTCTCAGAGCTTCAA‐3′	95°C, 5 min; 95°C, 30 s;		
	5′‐CGACACCACGGAGGAAGAGAAGA‐3′	59°C, 30 s; 72°C, 30 s;	BsrⅠ	229 bp
		38 cycles; 72°C, 10 min		
rs3761548	5′‐CCTCTCCGTGCTCAGTGTAG‐3′	95°C, 5 min; 95°C, 30 s;		
	5′‐GCCTCAGCCTTCGCCAATA‐3′	61°C, 30 s; 72°C, 30 s;	PstⅠ	473 bp
		36 cycles; 72°C, 10 min		
rs3761547	5′‐GCAATCCTCCTCTCGCACAC‐3′	95°C, 5 min; 95°C, 30 s;		
	5′‐TGCAGGGCTTCAAGTTGACAG‐3′	60°C, 30 s; 72°C, 30 s;	PvuIⅠ	183 bp
		36 cycles; 72°C, 10 min		
rs2280883	5′‐GGGTGTTACAAGGAAAGGTTGGGAC‐3′	95°C, 5 min; 95°C, 30 s;		
	5′‐ACCTAACCCTCTCCTGGACCCATA‐3′	61°C, 30 s; 72°C, 30 s;	MspⅠ	205 bp
		38 cycles; 72°C, 10 min		

Abbreviation: PCR‐RFLP, polymerase chain reaction‐restriction fragment length polymorphism.

### 2.4 Statistical analysis

The statistical analyses were performed with the SPSS Statistics version 17.0 (SPSS, Inc.). *χ* ^2^ test was used to assess significant differences in genotype and allele frequencies among the groups. The association of SNPs with GD risk was evaluated by odds ratios (ORs) with 95% confidence intervals (CIs) for five different genetic models. Statistical significance was established at *p* < .05. For controls, SNPStats (http://bioinfo.iconcologia.net/SNPStats/start. HTM) was used to analyze each SNP of Hardy‐Weinberg equilibrium (HWE).

## 3 RESULTS

### 3.1 Clinical features of the enrolled GD patients

The detailed demographic characteristics and clinical manifestations of the enrolled patients are shown in Supporting Information: Table S1. The study included 503 GD patients (male, 138; female, 365; mean age, 39.04 ± 14.31 years) and 890 healthy controls (male, 282; female, 608; mean age, 39.90 ± 12.24 years). There was no significant difference in age and sex between the control and GD groups (*p* > .05). In the control group, the genotype frequency distribution of the four studied SNPs (rs3761547, rs3761548, rs3761549, and rs2280883) did not deviate from the HWE.

### 3.2 Allele and genotype frequencies of the four SNPs in the first phase of the study

Considering that *FoxP3* gene is located on the X chromosome, we divided the patients into two groups, namely, female and male. A two‐stage case‐control study was conducted. In the first stage study, we tested the association between the four SNPs (rs3761547, rs3761548, rs3761549, and rs2280883) of *FoxP3* gene and GD susceptibility in 195 GD cases and 367 healthy controls. The results showed that significantly increased frequencies of A allele (*p* = .031, OR = 1.635) and AA genotype (*p* = .023, OR = 3.257) of *FoxP3/*rs3761548 were observed in female GD patients as compared to the healthy controls (Table 2). Moreover, as compared to the healthy male group, GD male patients had a lower frequency of *FoxP3*/rs3761548 A allele (*p* = .049, OR = 0.453) and had a higher frequency of *FoxP3*/rs3761548 C allele (*p* = .049, OR = 2.209) (Table 3). None of the other SNPs, including rs3761547, rs3761549 and rs2280883, were associated with GD susceptibility (*p*＞.05; Supporting Information: Table S2, Table S3).

**Table 2:** Allele and genotype frequencies of *FoxP3*/rs3761548 in female GD patients and healthy controls.

Gene	Allele/Genotype	GD patients (%)	Controls (%)	*p* Value	OR (95% CI)
rs3761548 (C＞A) Stage 1	A	50 (20.2%)	42 (13.4%)	**.031**	1.635 (1.044−2.563)
	AA	12 (9.7%)	5 (3.2%)	**.023**	3.257 (1.116−9.509)
	AC	26 (21%)	32 (20.4%)	.904	1.036 (0.580−1.853)
	CC	86 (69.4%)	120 (76.4%)	.183	0.698 (0.410−1.186)
	C	198 (79.8%)	272 (86.6%)	**.031**	0.611 (0.390−0.958)
rs3761548 (C＞A) Stage 2	A	96 (19.9%)	120 (13.3%)	**.0012**	0.617 (0.459−0.829)
	AA	11 (4.6%)	11 (2.4%)	.129	1.913 0.817−4.479
	AC	74 (30.7%)	98 (21.7%)	**.009**	1.596 (1.121−2.273)
	CC	156 (64.7%)	342 (75.8%)	**.002**	0.585 (0.416−0.823)
	C	386 (80.1%)	782 (86.7%)	**.0012**	1.621 (1.207−2.176)
rs3761548 (C＞A) Combined	A	146 (20%)	162 (13.3%)	**<.001**	1.672 (1.273−2.079)
	AA	23 (6.3%)	16 (2.6%)	**.005**	2.488 (1.297−4.775)
	AC	100 (27.4%)	130 (21.4%)	**.032**	1.388 (1.027−1.875)
	CC	242 (66.3%)	462 (76%)	**.001**	0.622 (0.467−0.828)
	C	584 (80%)	1054 (86.7%)	**<.001**	0.615 (0.481−0.786)

*Note*: Bold values indicates statistically significant.

Abbreviations: CI, confidence interval; OR, odds ratio.

**Table 3:** Allele frequencies of *FoxP3*/rs3761548 in male patients with GD and male healthy controls.

Gene	Allele/Genotype	GD patients (%)	Controls (%)	*p* Value	OR (95% CI)
rs3761548 (C＞A) Stage 1	A	8 (11.3%)	46 (21.9%)	**.049**	0.453 (0.202−1.013)
	C	63 (88.7%)	164 (78.1%)	**.049**	2.209 (0.988−4.941)
rs3761548 (C＞A) Stage 2	A	17 (25.4%)	15 (20.8%)	.525	1.292 (0.586−2.851)
	C	50 (74.6%)	57 (79.2%)	.525	0.774 (0.351−1.708)
rs3761548 (C＞A) Combined	A	25 (18.1%)	61 (21.6%)	.402	0.802 (0.478−1.345)
	C	113 (81.9%)	221 (78.4%)	.402	1.248 (0.743−2.094)

*Note*: Bold values indicates statistically significant.

Abbreviations: CI, confidence interval; OR, odds ratio. Bold values indicates statistically significant.

### 3.3 Allele and genotype frequencies of *FoxP3*/rs3761548 in the second‐stage and combined study

*FoxP3/*rs3761548 genotypes were identified in an additional 308 GD patients and 523 healthy controls to further validate the positive results of the phase first study. The results showed consistence with the first stage (A allele: *p* = .0012, OR = 0.617; C allele: *p* = .0012, OR = 1.621, respectively). In addition, a significant association of AC genotype (*p* = .009, OR = 1.596) and CC genotype (*p* = .002, OR = 0.585) of *FoxP3/*rs3761548 with female GD patients was observed (Table 2). However, there was no significant difference in frequencies of A and C allele of *FoxP3*/rs3761548 between the male patients with GD and the healthy controls. The combined data confirmed the association between *FoxP3*/rs3761548 and the female patients with GD (A allele: *p* < .001, OR = 1.672; AA genotype: *p* = .005, OR = 2.488; AC genotype: *p* = .032, OR = 1.388; CC genotype: *p* = .001, OR = 0.622; C allele: *p* < .001, OR = 0.615, respectively) (Table 3).

## 4 DISCUSSION

This study investigated the association between *FoxP3* gene polymorphism and GD in a Southwest Chinese Han population. It was found that there is a significant association between *FoxP3*/rs3761548 and female GD patients, while the rs3761547, rs3761549 and rs2280883 were not correlated to GD susceptibility. Based on the comparison between the female control group and the female GD group, we observed significant differences in the frequencies of *FoxP3*/rs3761548 A allele and AA genotype.

Among these four candidate polymorphisms (rs3761548, rs3761547, rs3761549, and rs2280883), only the association of rs3761548 with GD susceptibility in female GD patients was identified in the first stage of our study. It was demonstrated a loss of binding to E47 and c‐Myb factors occurred in patients with the rs3761548 AA genotype, leading to the reduced *FoxP3* transcription.^19^ Thus, the A allele of *FoxP3*/rs3761548 was demonstrated to affect the *FoxP3* expression and aggravate the severity of the immune response.^19^ From the perspective of clinical research, several studies showed that *FoxP3*/rs3761548 SNP is also associated with the development of other autoimmune diseases, such as psoriasis, Behcet's disease (BD), vitiligo, ulcerative colitis (UC), allergic rhinitis, rheumatoid arthritis (RA), multiple sclerosis (MS), Hashimoto's thyroiditis (HT).^20^, ^21^, ^22^, ^23^, ^24^, ^25^, ^26^, ^27^, ^28^ A recent meta‐analysis of susceptibility to various autoimmune diseases showed an association between rs3761548 and autoimmune disease.^29^ An additional recent meta‐analysis showed that the rs3761548 polymorphism of the *FoxP3* was associated with increased GD risk in Asians, due to the Tregs suppression and the enhancement of autoimmune responses.^30^ Similarly, our recent meta‐analysis also showed that rs3761548 was associated with GD in Asians in the subgroup analysis according to ethnicity.^31^

In the second stage of our study, we found that there were significant differences in the frequencies of the *FoxP3*/rs3761548 A allele, AA genotype, AC genotype, CC genotype and C allele between female healthy controls and GD patients. The *FoxP3*/rs3761548 polymorphisms the AA genotype was also reported to be a risk factor for several diseases, including MS, acute coronary syndrome, systemic lupus erythematosus, vitiligo, allergy and thyroid cancer.^27^, ^32^, ^33^, ^34^, ^35^, ^36^ It is found that the *FoxP3*/rs3761548 AA genotype was correlated with a significant decrease in the FoxP3 protein level in the Tregs of generalized vitiligo patients.^22^ Additionally, *FoxP3*/rs3761548 A allele has been identified to be significantly associated with BD, UC, MS, and RA.^21^, ^23^, ^37^, ^38^ It was demonstrated that the A allele of *FoxP3*/rs3761548 affected Tregs function, which is one of the factors involved in the susceptibility for MS in females.^26^ Earlier research has established a link between the *FoxP3*/rs3761548 and an elevated risk of severe osteoarthritis in the Turkish population.^39^ More recently, a study discovered that the C allele of the rs3761548 increased the susceptibility to HT.^28^ However, intriguingly, no significant difference was observed in the prevalence of rs3761548 between GD and control groups in both Indian and British populations.^16^, ^17^ Other research also indicated that there was no notable difference in the incidence of *FoxP3*/rs3761548 among healthy and GD groups in children and adolescents.^14^ These disparities in results could be attributed to the varying populations studied. Our study proposes that there may be differences in the frequency of *FoxP3*/rs3761548 among GD patients from diverse populations, suggesting it could be a risk factor for Asian GD patients. Regarding the other three *FoxP3* variations (rs3761549, rs3761547, and rs2280883), our data revealed no significant differences in allele and genotype frequencies between case and control groups, which was consistent with other previous studies.^12^, ^13^, ^14^, ^15^, ^16^, ^40^ However, previous research by Yu et al.^12^ and Zheng et al.^13^ demonstrated an association between the rs2280883 variant and GD susceptibility in both the Zhejiang Han and Hubei Han populations. A possible explanation for these inconsistent results is that the sample sizes in these studies were small and the research subjects were distributed in different regions of China.

Moreover, our combined study observed significant differences between the female control group and the female GD group in the frequencies of the *FoxP3*/rs3761548 A allele and AA genotype. In contrast, no significant correlation between *FoxP3*/rs3761548 and GD was found in the male population in the combined study. A deeper understanding of these findings can be achieved by examining the location of the FoxP3 gene on the X chromosome and its correlation with GD. Various potential mechanisms, including probabilistic and X‐inactivation, could account for the higher incidence of GD in women at the X chromosomal locus.^41^ As for the application value of our finding in clinical work, we believe that understanding the genetic basis of GD might help identify individuals at risk, which could guide early intervention and treatment decisions. The specific role of *FoxP3*/rs3761548 polymorphism in this regard requires further investigation.

Several limitations of our study need to be specified. First, we investigated only a limited number of SNPs in *FoxP3* gene. It is possible that there are other SNPs in *FoxP3* gene to be associated with GD susceptibility. Second, only Southwest Chinese Han population was enrolled, and it is not certain whether our findings can be generalized to other ethnic populations. Third, there are limited numbers of subjects in each subgroup according to gender and study stage, which might reduce the test power of this study. Furthermore, there are lack of functional study on the mechanism of *FoxP3/*rs3761548 polymorphism in regulating Tregs and Th17/Treg homeostasis.

## 5 CONCLUSION

In summary, our results suggested that *FoxP3*/rs3761548, but not the rs3761547, rs3761549 and rs2280883, is associated with an increased risk of GD in female patients in a Southwest Chinese Han population. Further studies are needed to elucidate the role of *FoxP3*/rs3761548 and Th17/Treg homeostasis in the development of GD.

## AUTHOR CONTRIBUTIONS

**Hongsong Yu**: Conceptualization, methodology, writing—review and editing, funding acquisition. **Xin Wang**: Investigation, resources, funding acquisition. **Guiqin Tan**: Investigation, writing—original draft. **Guangbing Zheng**: Investigation, formal analysis. **Jiang Li**: Validation. **Yingping Zhu**: Validation. **Zhongzhi Liang**: Validation. **Hua Li**: Resources.

## CONFLICT OF INTEREST STATEMENT

The authors declare no conflict of interest.

## ETHICS STATEMENT

The study protocol was conducted following the Declaration of Helsinki and approved by the Ethics Committee for Zunyi Medical University (2019‐H‐001) and the Yongchuan Hospital affiliated to Chongqing Medical University (2015‐11).

## Supporting information

Supplementary 1. Table S1. Clinical characteristics, age and gender distribution in controls as well as patients with GD. Supplementary 2. Table S2. Allele and genotype frequencies of *FoxP3* rs3761547, rs3761549 and rs2280883 between female patients with GD and female healthy controls. Supplementary 3. Table S3. Allele frequencies of *FoxP3* rs3761547, rs3761549 and rs2280883 SNPs between male patients with GD and male healthy controls.

## Data Availability

Data are available upon request from the authors.
