# Supplementary material for: Association of genetic variations in FoxP3 gene with Graves' disease in a Southwest Chinese Han population
Source: Immun Inflamm Dis. 2023 Oct 13;11(10):e1046. doi: 10.1002/iid3.1046 (PMC10571500; doi:10.1002/iid3.1046)
Supplement: Supplementary file 1 — Supplementary 1. Table S1. Clinical characteristics, age and gender distribution in controls as well as patients with GD. Supplementary 2. Table S2. Allele and genotype frequencies of FoxP3 rs3761547, rs3761549 and rs2280883 between female patients with GD and female healthy controls. Supplementary 3. Table S3. Allele frequencies of FoxP3 rs3761547, rs3761549 and rs2280883 SNPs between male patients with GD and male healthy controls. [file IID3-11-e1046-s001.docx]

**TABLE S1** Clinical characteristics, age and gender distribution in controls as well as patients with GD.

| Clinical manifestations | Total | Percentage (%) |
| --- | --- | --- |
| Patients with GD | 503 |  |
| Mean age [M±SD] | 39.04±14.31 |  |
| Male | 138 | 27.4 |
| Female | 365 | 72.6 |
| Hyperthyroidism | 503 | 100 |
| Diffuse thyroid enlargement | 503 | 100 |
| Graves' ophthalmopathy | 46 | 9.1 |
| Controls | 890 |  |
| Mean age [M±SD] | 39.90±12.24 |  |
| Male | 282 | 31.7 |
| Female | 608 | 68.3 |

**TABLE S2** Allele and genotype frequencies of *FoxP3* rs3761547, rs3761549 and rs2280883 between female patients with GD and female healthy controls.

| Gene | Allele/ Genotype | GD patients (%) | Controls (%) | *P Value* | *OR* (95 % CI) |
| --- | --- | --- | --- | --- | --- |
| rs3761547（A＞G） | A | 151(83%) | 253(80.6%) | 0.455 | 1.168(0.776-1.759) |
|  | AA | 63(69.2%) | 104(66.2%) | 0.433 | 1.212(0.750-1.959) |
|  | AG | 25(27.5%) | 45(28.7%) | 0.468 | 0.830(0.501-1.374) |
|  | GG | 3(3.3%) | 8(5.1%) | 0.841 | 0.899(0.318-2.543) |
|  | G | 31(17%) | 61(19.4%) | 0.455 | 0.856(0.569-1.288) |
| rs3761549（C＞T） | T | 37(20.3%) | 56(17.8%) | 0.864 | 0.966(0.648-1.440) |
|  | TT | 5(5.5%) | 8(5.1%) | 0.994 | 1.004(0.378-2.668) |
|  | TC | 27(29.7%) | 40(25.5%) | 0.826 | 0.946(0.575-1.555) |
|  | CC | 59(64.8%) | 109(69.4%) | 0.838 | 1.050(0.657-1.679) |
|  | C | 145(79.7%) | 258(82.2%) | 0.864 | 1.035(0.694-1.544) |
| rs2280883（C＞T） | T | 147(84.5%) | 272(86.6%) | 0.676 | 1.105(0.691-1.768) |
|  | TT | 61(70.1%) | 120(76.4%) | 0.943 | 1.019(0.603-1.722) |
|  | TC | 25(28.7%) | 32(20.4%) | 0.737 | 1.098(0.637-1.890) |
|  | CC | 1(1.1%) | 5(3.2%) | 0.259 | 0.397(0.076-2.080) |
|  | C | 27(15.5%) | 42(13.4%) | 0.676 | 0.905(0.566-1.447) |

OR: Odds ratio; CI: Confidence interval.

**TABLE S3** Allele frequencies of *FoxP3* rs3761547, rs3761549 and rs2280883 SNPs between male patients with GD and male healthy controls.

| Gene | Allele/Genotype | GD patients (%) | Controls (%) | *P* Value | OR (95 % CI) |
| --- | --- | --- | --- | --- | --- |
| rs3761548（C＞A）Stage 2 | A | 15(24.6%) | 15(20.8%) | 0.525 | 1.292(0.586-2.851) |
|  | C | 46(75.4%) | 57(79.2%) | 0.525 | 0.774(0.351-1.708) |
| rs3761548（C＞A）  Combined | A | 18(18%) | 61(21.6%) | 0.402 | 0.802(0.478-1.345) |
|  | C | 82(82%) | 221(78.4%) | 0.402 | 1.248(0.743-2.094) |
| rs3761547（A＞G） | A | 28(73.7%) | 161(82.5%) | 0.072 | 0.568(0.306-1.057) |
|  | G | 10(26.3%) | 32(17.5%) | 0.072 | 1.760(0.946-3.273) |
| rs3761549（C＞T） | T | 9(28.1%) | 30(14.4%) | 0.13 | 1.663(0.858-3.224) |
|  | C | 23(71.9%) | 179(85.6%) | 0.13 | 0.601(0.310-1.166) |
| rs2280883（C＞T） | T | 32(86.5%) | 165(78.6%) | 0.074 | 1.864(0.935-3.714) |
|  | C | 5(13.5%) | 45(21.4%) | 0.074 | 0.537(0.269-1.069) |

OR: Odds ratio; CI: Confidence interval.
